# Sharing simulation-based training courses between institutions: opportunities and challenges

**DOI:** 10.1186/s41077-016-0034-x

**Published:** 2017-01-06

**Authors:** Torrey A. Laack, Ellen A. Lones, Donna R. Schumacher, Frances M. Todd, David A. Cook

**Affiliations:** 1 Multidisciplinary Simulation Center, Mayo Clinic, Rochester, Minnesota USA; 2 Department of Emergency Medicine, Mayo Clinic, Rochester, Minnesota USA; 3 Dartmouth-Hitchcock Patient Safety Training Center, Lebanon, NH USA; 4 Division of General Internal Medicine, Mayo Clinic, Rochester, Minnesota USA

**Keywords:** Mayo Clinic, Target Audience, Moderate Sedation, Sharing Platform, Regular Team Meeting

## Abstract

**Background:**

Sharing simulation-based training (SBT) courses between institutions could reduce time to develop new content but also presents challenges. We evaluate the process of sharing SBT courses across institutions in a mixed method study estimating the time required and identifying barriers and potential solutions.

**Methods:**

Two US academic medical institutions explored instructor experiences with the process of sharing four courses (two at each site) using personal interviews and a written survey and estimated the time needed to develop new content vs implement existing SBT courses.

**Results:**

The project team spent approximately 618 h creating a collaboration infrastructure to support course sharing. Sharing two SBT courses was estimated to save 391 h compared with developing two new courses. In the qualitative analysis, participants noted the primary benefit of course sharing was time savings. Barriers included difficulty finding information and understanding overall course flow. Suggestions for improvement included establishing a standardized template, clearly identifying the target audience, providing a course overview, communicating with someone familiar with the original SBT course, employing an intuitive file-sharing platform, and considering local culture, context, and needs.

**Conclusions:**

Sharing SBT courses between institutions is feasible but not without challenges. An initial investment in a sharing infrastructure may facilitate downstream time savings compared with developing content de novo.

## Background

Simulation-based training (SBT) is widely used to train individuals and teams with a goal of improving quality of care provided and patient safety. Mounting evidence in the literature suggests that SBT is an effective training strategy for teaching technical and teamwork skills [1–3], and SBT is associated with superior learning outcomes as compared with other teaching modalities [4, 5]. However, these benefits come at a price, with the cost of SBT being a barrier to implementation [6]. Few studies have directly compared the costs of SBT against alternative educational strategies, and the amount of time and resources devoted to developing and maintaining simulation courses and curricula remain largely unquantified [7–9].

One proposed key to transforming medical education in the 21st century is to break down professional silos while enhancing collaboration and “linking together through networks, alliances, and consortia between educational institutions worldwide” [10]. Although every hospital, medical center, and clinic has unique challenges, institutional needs still have significant overlap. Often, the common needs could be addressed and solutions shared through collaboration and resource pooling, yet few collaborations for sharing SBT courses have been reported [11]. One US group reported successfully shared simulation-based assessment materials with colleagues in Israel [12]. Another group created a free library of simulation scenarios, but an evaluation of the library reported only download rates (which were high) and new contributions (which were low) [13]. The Association of American Medical Colleges’ MedEdPORTAL supports “the open exchange of peer-reviewed health education teaching and assessment resources” [14]. Although this repository is highly utilized and includes numerous simulation-based education resources, published evaluations of its usefulness are limited [15]. Additional collaborative ventures for simulation training, focusing primarily on faculty development and joint development of new courses, have emerged across regions of North America, including California, Texas, Minnesota, Alberta, and British Columbia [16–22].

A chief advantage of sharing is the anticipated cost savings achieved by reducing faculty time for de novo course development. In addition, the process of sharing SBT courses could lead to the development of a community of individuals with similar interests and subsequent advances in course content and research. However, important challenges may also be encountered when attempting to apply previously developed SBT content at another institution. Thus, although the sharing of simulation-based educational resources theoretically should work, how well this process really works and what barriers are encountered during implementation remain unknown.

The purpose of the present study was to evaluate the process of sharing established simulation-based courses across institutions and to identify areas for improvement in future sharing activities.

## Methods

### Overview

In 2013, simulation centers at Mayo Clinic (Rochester, Minnesota) and Dartmouth-Hitchcock Medical Center (D-H; Lebanon, New Hampshire) formally established a collaborative network to share SBT courses across institutions. The current project evaluated this collaboration, with the long-term vision of continued sharing between D-H and Mayo Clinic and with other institutions as well. Considerable time and resources went into building this collaboration, which included preparing an infrastructure to inventory SBT courses, identifying content ready for sharing, and implementing sharing of specific SBT courses between sites.

We evaluated the process of sharing a total of four courses (two from each institution, shared with the other institution) by using quantitative and qualitative data including estimates of time spent developing and implementing each course, a survey of instructors involved in implementing shared courses, and 1-on-1 instructor interviews. The Mayo Clinic Institutional Review Board and the Dartmouth-Hitchcock Committee for the Protection of Human Subjects deemed the study exempt.

### Process and procedures for sharing

Each site assembled a local team of simulation leaders, SBT educators, and administrators who met regularly to review the curricula, identify courses that could be shared, and monitor the process and progress of sharing. A shareability matrix was developed to define course readiness for sharing and to give potential users information about the course. At each site, an individual with training and experience in simulation and education scored every available course using the parameters of frequency, maturity, and completeness on a scale of 1 to 5, with 1 being least ready and 5 being most ready. Based on this scoring, an inventory of courses ready for sharing was identified.

The project teams chose two courses from each institution to share. For the first exchange, each team identified an existing SBT course for the same clinical task, “Moderate Sedation” for nurses, to contrast different approaches to the same topic and to focus on the process of sharing. Each organization provided their course to the other to implement. For the second course exchange, leaders at each site reviewed the other site’s inventory of shareable courses to identify a SBT course that would fulfill a known institutional need not already covered in their own institution’s training inventory. Mayo Clinic implemented the D-H “Stroke and Seizure” course for nurses and D-H implemented the Mayo Clinic “Central Line Workshop” for physicians. Instructors at each site with experience in SBT and appropriate content expertise implemented each shared course. Institutions followed local best practices when specific course elements were not explicitly specified.

Teaching methods used in the courses included slideshow presentations, scenarios using high-fidelity patient simulators, supporting articles, procedural task trainers, quizzes, and electronic learning content. A web-based file-sharing application provided a centralized repository for individuals at each site to share and access SBT materials.

### Outcomes and data collection

Participants providing outcomes for this study were course instructors at Mayo Clinic and D-H. Sample size calculations were not conducted because the sample was determined by the number of instructors involved. Outcomes focused on instructor experiences with implementation of shared courses and faculty time needed for development and implementation of SBT courses. We evaluated designated outcomes after the course using 1-on-1 interviews and a written survey questionnaire.

Qualitative data were obtained from two sources. First, each questionnaire included open-ended questions regarding barriers, enabling factors, and suggestions for improvement. Second, each instructor participated in a 1-on-1 semi-structured interview with an investigator (E.A.L. or D.R.S.) using a flexible interview template. Interviewers asked about context-specific factors that made implementation easier or harder, important omissions in shared material, and suggestions for improvement. Interviews were transcribed for subsequent analysis.

The primary quantitative outcome was overall cost in terms of time. Each member of the implementation team and each instructor retrospectively estimated the total time spent organizing the exchange process, preparing to share a course, and implementing a shared course. Developers of original course plans and materials also retrospectively estimated the time spent creating the Central Line Workshop and Stroke and Seizure courses, but time estimates were not available for the Moderate Sedation courses. We did not convert time to monetary units because we anticipated variability in staff roles and salary ranges across institutions and because precise salary information was considered confidential by both institutions.

Other quantitative outcomes included instructor perceptions regarding efficiency, effectiveness, time required to develop and deliver the course, relevance of course materials to local needs, and barriers to implementation. We identified specific items used to measure these outcomes through informal discussions with the project team members and course instructors. Based on these items, we created a questionnaire that asked instructors to compare the shared course (and the experience of implementing the course) with locally developed courses that they had led previously. We refined items for the final questionnaire through an iterative review process among the project leadership team, and pilot testing on two simulation instructors not engaged in the course sharing implementation.

### Data analysis

We performed a qualitative analysis of the interview transcripts and responses to open-ended survey questions with the intent of identifying specific changes to improve the sharing process. We used the constant comparative method [22] by first identifying strengths and weaknesses of the shared-course approach, and then contrasting these strengths and weaknesses to define key factors influencing the course-sharing process. We supplemented this analysis by identifying and grouping keywords in context, from which we formed thematic categories of comments. Finally, we integrated the key factors and thematic categories to create a grounded theory model explaining how sharing could be improved in future iterations [23–25].

Course evaluation and study-specific survey data were reported in aggregate using both mean (SD) and median (interquartile range) because of the small sample and because the data did not follow a normal distribution.

## Results

### Instructor characteristics

All instructors involved in the implementation of shared courses (eight nurses, one physician, and one allied health professional; five from each site) were surveyed and interviewed. All had prior simulation experience, and half had previously taught at least ten simulation-based courses.

### Survey results

Responses from the instructor survey are shown in Table 1. Most ratings suggested a neutral impression (median score of 4) regarding delivering the course, relevance of course objectives, and use of key resources and assets. Instructors believed that the course-sharing approach was more efficient and that materials were more complete compared with courses developed de novo. However, instructors perceived more barriers to preparing and delivering the shared courses and thought that content and assessments were less relevant to local needs.

**Table 1:** Instructor perceptions regarding the implementation of shared courses (*N*=10)

How did this shared course compare with developing and delivering a local simulation-based course, in terms of …	Mean (SD)	Median (IQR)
Overall course efficiency (effectiveness per instructor time invested)	4.9 (1.7)	5 (4–6)
Overall course educational effectiveness	4.4 (1.8)	4 (4–6)
Time spent preparing (planning, pilot-testing, refining) the course	5.1 (1.7)	6 (4–6)
Time spent delivering the course	4.2 (1.6)	4 (4–4)
Barriers/challenges in preparing the course	3.6 (1.7)	4 (2–5)
Barriers/challenges in delivering the course	3.5 (1.3)	4 (3–4)
Relevance of the course to this specific learner group	3.8 (1.8)	4 (3–4)
Relevance of the course objectives to local needs and clinical practice	3.8 (1.6)	4 (3–4)
Relevance of the course content to local needs and clinical practice	3.5 (1.7)	3.5 (2–4)
Relevance of the course assessment to local needs and clinical practice	3.7 (1.8)	4 (2–4)
Completeness of the course as outlined in curriculum materials	4.8 (1.8)	5 (4–6)
Availability of key resources (e.g., rooms, simulators, materials, support staff)	4.0 (1.4)	4 (4–4)
Preparing/training simulation assets (e.g., programming mannequins, training standardized patients, preparing task models)	3.8 (1.0)	4 (4–4)
Problems with simulation assets	3.7 (0.9)	4 (4–4)

All course instructors were surveyed using the items above; responses used a 7-point scale, with anchors of “1 = local much better” and “7 = shared much better”

*IQR* interquartile range

### Analysis of time investment

Considerable time and resources went into building this collaboration. Table 2 details the estimated time dedicated to the collaboration, including time spent on course inventory and appraisal of shareability, development of a web-based sharing platform, a site visit to each institution, and regular team meetings to assess the progress of the collaboration.

**Table 2:** Estimated total time invested in collaboration

Personnel	Time, h		
	Mayo	D-H	Total
Project or operations managers	107	43	150
Simulation center directors	69	80	149
Education specialists	129	132	261
Information technology support	24	34	58
Total	329	289	618

*D-H* Dartmouth-Hitchcock Medical Center, *Mayo* Mayo Clinic

Time required to implement a shared course vs develop the same course de novo is shown in Table 3. The Mayo team translated the D-H Stroke and Seizure course into Mayo's established scenario template format prior to use, which accounts for the relatively large amount of time for the implementation process (51 h (50% of the original D-H development time)). In contrast, D-H implemented Mayo Clinic’s Central Line Workshop in its original format, and it thus required substantially less implementation time (9 h (2.6% of Mayo Clinic development time)).

**Table 3:** Time estimates for de novo development vs implementation of shared courses

Course	Time, h		
	De novo course development	Implementation of shared course	Time saved
Central Line Workshop	350 (Mayo)	9 (D-H)	341
Stroke and Seizure	101 (D-H)	51 (Mayo)	50

*D-H* Dartmouth-Hitchcock Medical Center, *Mayo* Mayo Clinic

### Qualitative analysis: benefits of sharing curricula

Analysis of free-text comments and interview transcripts identified several themes that will guide future sharing activities. We noted two themes relevant to the perceived benefits of sharing. First, participants recognized a significant benefit from time savings.

“… it was faster to tweak it than it was to start from scratch and write that course.” (Mayo)

“There was very little additional work that needed to be done in order to implement the course.” (D-H)

“It is always nice to not have to start with a blank page!” (Mayo)

Second, participants noted a general efficiency in implementing already-written scenarios.

“I think the concept and the idea of sharing is a wonderful thing, I mean, why reinvent the wheel?” (D-H)

### Qualitative analysis: tips for success in sharing curricula

We also identified several barriers, including difficulty finding important information needed to run the course, an unclear target audience, and lack of understanding of overall course flow. Participants also noted that local practice patterns may require a course to be modified prior to sharing across diverse settings.

Through an in-depth analysis of the participant narratives surrounding these barriers, we generated a preliminary grounded theory model suggesting six actions that will improve the chances of success in future curricular sharing efforts: (1) establish a standardized template, (2) identify the target audience, (3) provide a course overview, (4) designate a contact person at the sharing site, (5) use an intuitive sharing platform, and (6) consider local culture, context, and needs. These points are elaborated with supportive quotes in Table 4.

**Table 4:** Key elements for success in course sharing

Theme	Detail	Comment
Establish and use a standardized template	A common concern was the absence of a consistent template to organize course information Information typically was available but cumbersome to find and often in an unfamiliar format	“…having more of a standardized template because I didn’t have that one source to go for all my information.” (D-H) “I found that using two very different scenario templates to be quite problematic.” (Mayo) “I found myself looking through and opening every folder they had sent just to make sure I didn’t miss something. I wasn’t sure that this was the complete set of supplies.” (D-H)
Identify the target audience	Learner level was frequently unclear Because learner level was considered vital for preparing and modifying shared curricula, this was recommended as a crucial element in a standardized template	“I was not sure regarding who the course was structured for. For example, was this course for nursing students, novice nurses, or for more experienced nurses?” (Mayo) “I think the course would need to be modified depending upon what student population you were working with.” (D-H)
Provide a course overview	Overall course flow and specific equipment needs were unclear An agenda, overview of the course, and more detailed objectives could improve course flow and cadence Key decisions could be facilitated by listing required resources (eg, have a living standardized patient if the site’s mannequin cannot reproduce required symptoms)	“I did not feel that I had a good idea of the actual intent of the course… an overview or bird's eye picture.” (Mayo) “I think the material they had for us was good, but I think it would have been beneficial to have seen someone run through a full course start to finish to understand exactly what they were trying to convey.” (D-H)
Designate a contact person	For both sites, questions frequently arose when implementing a shared course, but no contact person on the developing team was indicated Implementation would be facilitated by ready access to an individual knowledgeable about the course	“I did not have a phone number or contact person…in order to clarify questions that I had or that the clinical nurses had.” (Mayo) “It wouldn’t hurt to have somebody who had run that program talk in a phone conference… I would be able to touch base with a [simulation] specialist or whoever kind of organized that program at the other institution… that would be very helpful to me.” (D-H)
Use an intuitive sharing platform	Many noted challenges with the document-sharing software Information was difficult to access An easy-to-use document-sharing infrastructure would substantially facilitate the course-sharing process	“I think the platform we use to share the information was cumbersome…that in and of itself I found time consuming and difficult to navigate.” (D-H)
Consider local culture, context, and needs	Practices and requirements may differ between institutions and practice sites Accommodating these differences required only modest adjustments to the course	“…I don’t think there was really anything that was difficult. I think we just had to recognize where the practice, our own individualized practice here, is slightly different…” (Mayo) “It was very easy, it went as smooth as could be; it’s our standard practice.” (D-H)

*D-H* Dartmouth-Hitchcock Medical Center, *Mayo* Mayo Clinic

## Discussion

We report successfully sharing four SBT courses between two academic institutions. The time required to implement a shared course appears to be less than the time required to independently develop SBT courses, and this efficiency was perceived by instructors as the primary advantage to course sharing. However, a large initial investment of time was needed to develop the course-sharing infrastructure and many barriers were identified. While there are potential benefits with sharing SBT courses, our study demonstrates that sharing of content between institutions is not as simple as it may at first appear. Suggestions to improve the sharing process include use of a standardized template, clearly defining the target audience, providing a course overview, having someone experienced with the specific SBT course available to contact for questions, adopting a user-friendly sharing platform, and consideration of local needs.

If cross-site collaboration is anticipated, development of a shared template should be considered. A well-designed template could clarify the target audience, provide a course overview, and have contact information for questions that may arise when attempting to implement shared content. Many templates are currently in use, and attempts have been made to improve this tool for SBT [26]. However, an agreed upon standard template for use across all disciplines and institutions remains elusive.

An efficient, user-friendly, and secure tool for electronic document sharing is also essential. Institutional requirements for secure document-sharing platforms limited our options during this study with many users finding the cumbersome password and permissions process and limited accessibility to the content a significant barrier; we have yet to identify a platform that meets the requirements of both security administrators and end-users.

Our study was limited by the small sample size (only ten faculty members were involved) and involved two institutions with a similar culture and similar educational resources. Sharing will likely be more difficult if culture, language, or learning environment differ. In addition, the estimates for development and implementation time were self-reported and subject to recall bias. This bias was likely greater for the initial development estimate compared with the implementation estimate because development occurred farther in the past (months to years vs weeks). We did not obtain time estimates for implementation or development of the Moderate Sedation courses because each site already had developed its own version, nor did we obtain data from the students enrolled in these courses because this was not the focus of our collaboration (although the Central Line Workshop has been evaluated previously) [27, 28]. The estimate of total time and time savings are likely highly variable across institutions, and will probably vary even within institutions for different courses. It is possible that a much less formal collaboration could have resulted in successful sharing of curricula. The qualitative analysis was limited by the quantity and depth of raw data available, yet strengthened by the iterative review of all available data by several members of the research team, reporting of supportive quotes, and proposal of specific, pragmatic tips for success in future curricular sharing efforts.

Although previous studies have described collaboration to develop new curricula [16–21], we are not aware of a similar study describing direct sharing of existing SBT courses between institutions. Publishing course descriptions and materials in venues such as MedEdPORTAL and peer-reviewed journals is appropriate for some content but requires a formal submission process that many instructors will not pursue. Further, while not specifically studied, we anticipate similar barriers to implementation of shared content from these sources (e.g., unfamiliar template, difficulty understanding overall course flow). Our goal was to enlarge the inventory of available SBT courses ready for sharing at our institutions without adding additional burdens on authors. The scoring system we used to determine which courses were most ready for sharing has not been formally evaluated, and was performed by only one person at each institution. Further research is needed to determine how well this or other scoring systems identify courses that can be easily and successfully shared.

The current financial climate of health care provides a growing incentive to decrease costs and work differently to improve efficiency. Our data show that instructors view potential time savings as the single biggest advantage to implementing a SBT course developed at another institution. This successful sharing process might have shown greater time savings had we extended it to additional courses. The initial formation of a cross-institutional collaboration required substantial resources that offset some of the benefits of decreased faculty time in the present study. However, it is possible that the large up-front cost of this investment can be amortized across future shared courses, with a much lower expense required to maintain the existing collaboration infrastructure. This is analogous to the initial cost of building a modern simulation center—it represents a 1-time expense that is spread over years of subsequent SBT courses. Finally, specific barriers identified can be addressed which should improve the efficiency and ease of future curricula sharing.

Additional advantages of sharing SBT courses exist independent of the potential time savings. Collaboration allows the opportunity to improve and customize existing courses. Identifying faculty with shared interests across sites creates a potential network of future collaborators for course development and research. In addition, sharing courses disseminates one’s work to a wider audience, which may in turn count toward academic promotion.

## Conclusions

The opportunity for cost avoidance through reduced course development time and reduced physician, nursing, and other staff time is a compelling motivator for sharing of SBT courses, but sharing is not as simple as it may at first appear. Our data suggest a reduction in development time with sharing, but this benefit was partially offset by the time and resources invested to generate a model for sharing and create and maintain the cross-institutional collaboration. Many barriers identified thus far appear to be largely avoidable with proper planning. Further research is needed to demonstrate whether sharing through a less formal process can yield similar results and whether incorporating the suggestions for improvement will further reduce time and streamline processes to implement shared SBT courses.

## Abbreviations

D-H: Dartmouth-Hitchcock Medical Center
SBT: Simulation-based training
